# Outcomes of second‐line antiretroviral therapy among children living with HIV: a global cohort analysis

**DOI:** 10.1002/jia2.25477

**Published:** 2020-04-15

**Authors:** The Collaborative Initiative for Paediatric HIV Education and Research (CIPHER) Global Cohort Collaboration, Kunjal Patel, Colette Smith, Intira Jeannie Collins, Ruth Goodall, Elaine J Abrams, Annette H Sohn, Thahira J Mohamed, Russell B Van Dyke, Pablo Rojo, Kara Wools‐Kaloustian, Jorge Pinto, Andrew Edmonds, Irene Marete, Mary Paul, Harriet Nuwaqaba‐Biribonwoha, Valériane Leroy, Mary‐Ann Davies, Rachel Vreeman, Nicky Maxwell, Venessa Timmerman, Charlotte Duff, Lynne Mofenson, Linda‐Gail Bekker, Marissa Vicari, Shaffiq Essajee, Martina Penazzato, Intira Jeannie Collins, Kara Wools‐Kaloustian, Mary‐Ann Davies, Valériane Leroy, Ruth Goodall, Kunjal Patel, Colette Smith, Rachel Vreeman, Amy Slogrove, Paige Williams, Siobhan Crichton, George Seage, Lineo Thahane, Peter N Kazembe, Bhekumusa Lukhele, Lumumba Mwita, Adeodata Kekitiinwa‐Rukyalekere, Sebastian Wanless, Mogomotsi S Matshaba, Intira Jeannie Collins, Ruth Goodall, Colette Smith, Tessa Goetghebuer, Claire Thorne, Josiane Warszawski, Luisa Galli, Sybil Geelen, Diana M Gibb, Carlo Giaquinto, Magdalena Marczynska, Laura Marques, Filipa Prata, Luminita Ene, Liubov Okhonskaia, Pablo Rojo, Antoni Noguera‐Julian, Lars Naver, Christoph Rudin, Gonzague Jourdain, Ali Judd, Alla Volokha, Jorge Pinto, Vanessa Rouzier, Regina Succi, Annette H Sohn, Azar Kariminia, Thahira J Mohamed, Marcel Yotebieng, Andrew Edmonds, Patricia Lelo, Rita Lyamuya, Irene Marete, Patrick Oyaro, Kara Wools‐Kaloustian, Andrew Boulle, Kennedy Malisita, Geoffrey Fatti, Andreas D Haas, Mary‐Ann Davies, Sophie Desmonde, Fatoumata Dicko, Valériane Leroy, Mark J Abzug, Paige Williams, Murli Purswani, George Seage, Russell Van Dyke, Ellen Chadwick, Elaine Abrams, Chloe Teasdale, Harriet Nuwagaba

**Affiliations:** ^1^ Department of Epidemiology Harvard T.H. Chan School of Public Health Boston MA USA

**Keywords:** children, perinatal HIV, second‐line, antiretroviral therapy, mortality, outcomes

## Abstract

**Introduction:**

Limited data describe outcomes on second‐line antiretroviral therapy (ART) among children globally. Our objective was to contribute data on outcomes among children living with HIV after initiation of second‐line ART in the context of routine care within a large global cohort collaboration.

**Methods:**

Patient‐level data from 1993 through 2015 from 11 paediatric HIV cohorts were pooled. Characteristics at switch and through two years of follow‐up were summarized for children who switched to second‐line ART after starting a standard first‐line regimen in North America, Latin America, Europe, Asia, Southern Africa (South Africa & Botswana) and the rest of sub‐Saharan Africa (SSA). Cumulative incidences of mortality and loss to follow‐up (LTFU) were estimated using a competing risks framework.

**Results:**

Of the 85,389 children on first‐line ART, 3,555 (4%) switched to second‐line after a median of 2.8 years on ART (IQR: 1.6, 4.7); 69% were from Southern Africa or SSA and 86% of second‐line regimens were protease inhibitor‐based. At switch, median age was 8.4 years and 50% had a prior AIDS diagnosis. Median follow‐up after switch to second‐line ranged from 1.8 years in SSA to 5.3 years in North America. Median CD4 counts at switch to second‐line ranged from 235 cells/mm^3^ in SSA to 828 cells/mm^3^ in North America. Improvements in CD4 counts were observed over two years of follow‐up, particularly in regions with lower CD4 counts at second‐line switch. Improvements in weight‐for‐age z‐scores were not observed during follow‐up. Cumulative incidence of LTFU at two years was <5% in all regions except SSA (7.1%) and Southern Africa (7.4%). Risk of mortality was <3% at two years of follow‐up in all regions, except Latin America (4.9%) and SSA (5.5%).

**Conclusions:**

Children switched to second‐line ART experience CD4 count increases as well as low to moderate rates of LTFU and mortality within two years after switch. Severe immune deficiency at time of switch in some settings suggests need for improved recognition and management of treatment failure in children.

## 1 Introduction

In 2018, there were an estimated 1.7 million children living with HIV globally and 160,000 new paediatric infections [1]. With the recommendation for immediate antiretroviral therapy (ART) [2], substantial gains in survival have been observed among children living with HIV [3]. The success of ART, however, brings new challenges, including the need to optimize regimens throughout the lifespan as children age into adolescence and adulthood. Evaluating critical ART outcomes such as HIV disease progression, mortality and loss to follow‐up (LTFU) is needed to inform treatment management strategies to increase long‐term effectiveness, as well as to help forecast future antiretroviral drug needs.

Responses to first‐line ART among children have been evaluated in large randomized trials and observational studies [4, 5, 6, 7, 8, 9, 10, 11, 12]. Studies evaluating outcomes on second‐line ART in children; however, have been limited by small sample sizes in both resource‐rich and resource‐limited settings [6, 13, 14, 15, 16, 17, 18, 19, 20, 21]. Of the four published studies on second‐line ART with larger sample sizes, ranging from 111 to 277 children, three were conducted in Thailand or the Asia‐Pacific region [18, 19, 20], and one followed Ugandan children who were switched to a lopinavir/ritonavir (LPV/r)‐based second‐line regimen [21]. These studies observed improvements in CD4 counts after switch to a second‐line regimen, with an increase of 267 cells/mm^3^ in mean CD4 counts after 48 weeks of follow‐up in the Ugandan study [21], and 463 cells/mm^3^ in median CD4 counts after 3.3 years of follow‐up in the largest study conducted in Asia [20]. No deaths were observed over 48 weeks of follow‐up among the Ugandan children on second‐line ART, and less than 2% of children died in the studies of Asian children on second‐line ART [18, 19, 20, 21].

The studies described above were conducted while experience with second‐line ART among children was still limited in many settings. The Collaborative Initiative for Paediatric HIV Education & Research (CIPHER) global cohort collaboration provides a unique opportunity to further evaluate outcomes associated with second‐line ART in the context of routine care, as it reflects the world’s largest combined cohort of children living with HIV on ART.

## 2 Methods

### 2.1 Study population

The CIPHER cohort collaboration brings together existing paediatric HIV observational cohorts to address key knowledge gaps in the care and treatment of children and adolescents living with HIV globally. Eleven international networks contributed data from 1993 through 2015 from a total of 47 countries: Baylor International Pediatric AIDS Initiative (BIPAI), European Pregnancy and Paediatric HIV Cohort Collaboration (EPPICC), the International epidemiology Databases to Evaluate AIDS (IeDEA) Consortium (Regions: Asia‐Pacific, Caribbean, Central and South America network (CCASAnet), Central Africa, East Africa, West Africa and Southern Africa), International Maternal Pediatric Adolescent AIDS Clinical Trials (IMPAACT) Protocols P219C and P1074, Optimal Models (ICAP at Columbia University), and the Pediatric HIV/AIDS Cohort Study (PHACS). All participating networks received local ethics approvals to transfer anonymized individual participant‐level data for this collaboration to the University of Cape Town (Cape Town, South Africa) for data management using a standardized protocol. The pooling of data at the University of Cape Town was approved by the University of Cape Town Health Research Ethics Committee (UCT HREC (reference 264/2014)). The dataset was then sent to University College London (London, United Kingdom) for analysis.

Eligible children were less than 10 years of age at cohort enrolment as a proxy for perinatal HIV infection, were less than 18 years of age at initiation of a “standard” first‐line combination ART regimen, and were seen in clinic for at least one visit after initiating ART. Children documented as horizontally infected with HIV (e.g. by blood products, unsafe injections, sexual transmission) and those enrolled in clinical trials of treatment monitoring, switch or interruption strategies were excluded.

### 2.2 Study definitions

“Standard” combination ART was defined as a regimen with at least three drugs, including at least two nucleoside/nucleotide reverse transcriptase inhibitors (NRTIs) plus either a non‐nucleoside reverse transcriptase inhibitor (NNRTI) or a ritonavir‐boosted protease inhibitor (PI). Switch to second‐line was defined as: (i) change of at least one NRTI plus either change in drug class (NNRTI to PI, or vice versa) or PI change; (ii) change from single to dual PI; or (iii) addition of a new drug class. Second‐line regimens including more than two drug classes were hierarchically classified based on whether they followed an NNRTI‐ or PI‐based first‐line ART. If first‐line was an *NNRTI‐based ART regimen*, the hierarchy was integrase strand transfer inhibitor (INSTI)>LPV/r>Other PI>efavirenz (EFV)>Other NNRTI. If first‐line was a *PI‐based ART regimen*, this was INSTI>EFV>nevirapine (NVP)>LPV/r>Other PI>Other NNRTI.

Geographical region was categorized as: North America, Latin America (Caribbean, Central and South America), Europe, Asia, Southern Africa (South Africa and Botswana) and the rest of sub‐Saharan Africa (SSA). Southern Africa was considered separately from SSA because this region had earlier introduction of LPV/r‐based regimens as first‐line and earlier rollout of viral load (VL) monitoring. Immunologic classification of HIV disease was based on World Health Organization (WHO) guidelines, and AIDS was defined as a WHO Stage 3/4 or a Centers for Disease Control and Prevention (CDC) Stage C clinical diagnosis [22, 23, 24]. Treatment monitoring strategy was derived from the frequency and availability of CD4 and VL measures across all children receiving ART within a cohort, as described previously [12].

### 2.3 Study outcomes

Outcomes of interest included CD4 counts and weight‐for‐age z‐scores (WAZ) at one and two years of follow‐up after switch to second‐line ART. Follow‐up was limited to two years based on the average duration of available follow‐up in SSA after switch to second‐line ART. Changes in CD4 counts and WAZ from switch to second‐line ART were also summarized. The British 1990 growth reference centiles were used to transform weight measures to WAZ for all regions due to the availability of standards across all age ranges [25]. Additional outcomes of interest included mortality and LTFU at one and two years of follow‐up. Children were considered as LTFU if they had no visit for more than one year (or more than two years for the North American and European cohorts due to annual reporting) before the last observed visit in their cohort. Children who met the definition of LTFU were censored at their last clinic visit.

### 2.4 Statistical analyses

This analysis was an intent‐to‐treat analysis, meaning that children were not excluded from analyses due to subsequent discontinuation or change of regimen after initial switch to second‐line ART when summarizing outcomes. Demographic and clinical characteristics at start of second‐line ART as well as follow‐up duration were summarized by region. Clinical and laboratory outcomes, including CD4 counts and WAZ, at one and two years after start of second‐line ART were then compared descriptively by region. CD4 counts and WAZ were additionally compared descriptively over follow‐up by CD4 count at switch, age at switch, WAZ at switch and second‐line regimen type. Summary statistics included only those with available data. The proportion of children with missing measures, however, was noted. Cumulative incidences of mortality and LTFU at one and two years of follow‐up were estimated using a competing risks framework [26] with LTFU treated as a competing risk for mortality, and vice versa. Transfer out was considered a competing risk for both outcomes. Stata version 14.2 (College Station, TX) was used to conduct all analyses.

## 3 Results

### 3.1 Study population characteristics at start of second‐line ART

**Figure 1:**
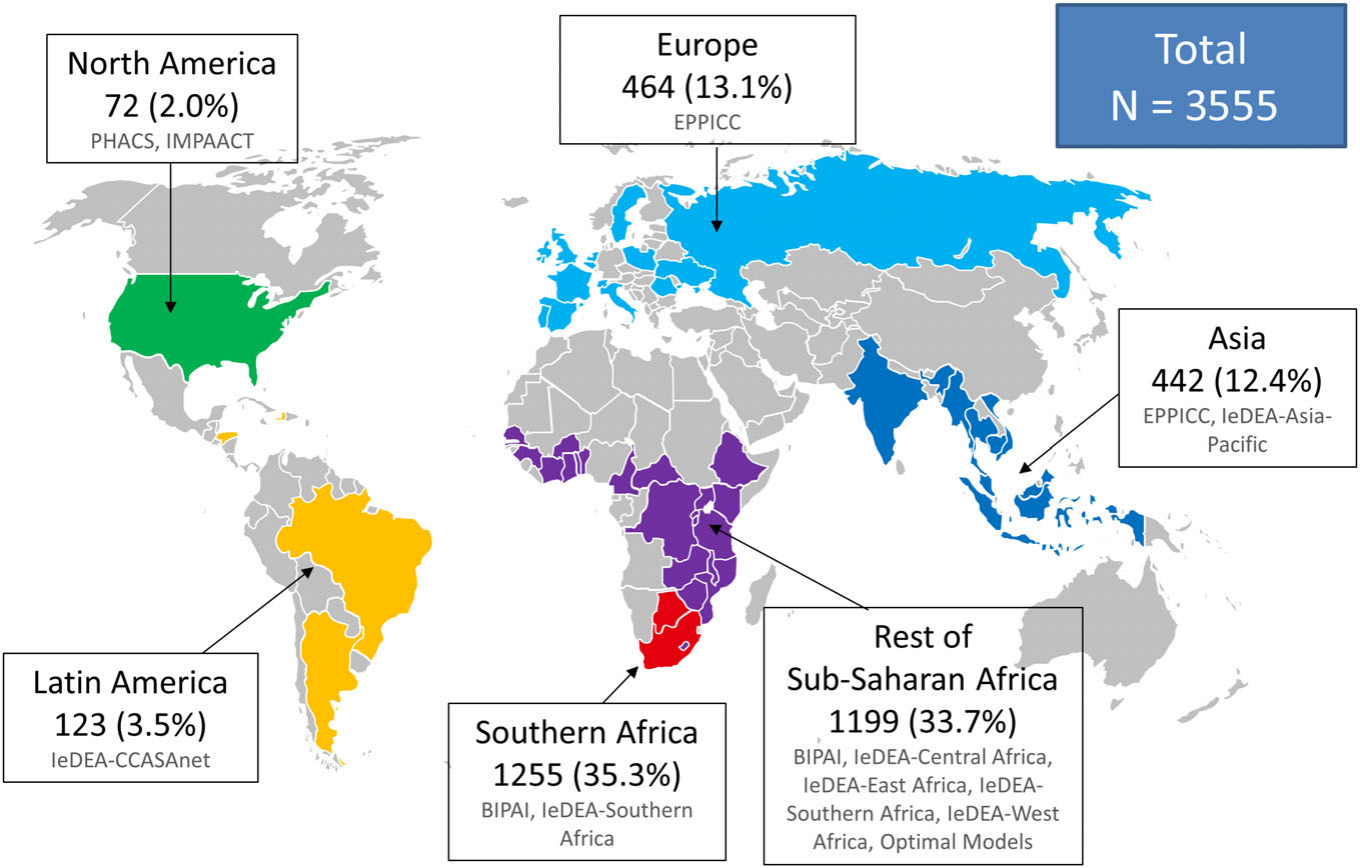
Global distribution of study population on second‐line ART (N = 3555).

Of over 170,000 children included in the CIPHER global cohort collaboration from the 11 participating networks, 85,389 (50%) initiated a “standard” first‐line ART regimen between 1993 and 2015 and were followed for switch to second‐line. Of these, 3,555 (4%) initiated a second‐line regimen, and outcomes were assessed over two years of follow‐up from the time of switch (Figure S1). Children from Southern Africa and SSA accounted for almost 70% of the overall study population, with an additional 13% from Europe, 12% from Asia, 3% from Latin America and 2% from North America (Figure 1). Table 1 shows demographic and clinical characteristics of the study population at start of second‐line ART. Forty‐four percent of the study population was female and 73% of children initiated second‐line ART between 2007 and 2012. Children who switched to second‐line ART spent a median of 2.8 years (interquartile range (IQR): 1.6, 4.7) on first‐line ART before switching; time on first‐line ART ranged from a median of 2.0 years in North America to 3.7 years in SSA. Median age at start of second‐line ART was 8.4 years (IQR: 5.3, 11.4), though there was regional variability with children in North America starting second‐line at earlier ages (median (IQR): 4.1 (1.9, 7.5) years).

**Table 1:** Demographic and clinical characteristics of children at the start of second‐line ART within the CIPHER cohort collaboration (N = 3555)

	North America	Latin America	Europe	Asia	Southern Africa	Rest of SSA	Total
Total N	72	123	464	442	1255	1199	3555
Median age in years (IQR)	4.1 (1.9, 7.5)	10.3 (6.7, 13.8)	8.2 (4.2, 12.0)	7.1 (5.2, 9.5)	8.3 (5.3, 11.2)	9.3 (6.0, 11.9)	8.4 (5.3, 11.4)
Female sex, N (%)	39 (54.2)	62 (50.4)	241 (51.9)	169 (38.2)	553 (44.1)	500 (41.7)	1564 (44.0)
Calendar year, N (%)							
Up to 2000	24 (33.3)	0 (0.0)	12 (2.6)	0 (0.0)	0 (0.0)	0 (0.0)	36 (1.0)
2001 to 2003	14 (19.4)	9 (7.3)	42 (9.1)	0 (0.0)	7 (0.6)	0 (0.0)	72 (2.0)
2004 to 2006	14 (19.4)	19 (15.5)	113 (24.4)	67 (15.2)	119 (9.5)	38 (3.2)	370 (10.4)
2007 to 2009	8 (11.1)	42 (34.2)	130 (28.0)	145 (32.8)	360 (28.7)	246 (20.5)	931 (26.2)
2010 to 2012	9 (12.5)	49 (39.8)	137 (29.5)	203 (45.9)	583 (46.5)	688 (57.4)	1669 (47.0)
2013 to 2015	3 (4.2)	4 (3.3)	30 (6.5)	27 (6.1)	186 (14.8)	227 (18.9)	477 (13.4)
Median years on first‐line ART (IQR)	2.0 (1.0, 3.5)	3.5 (2.2, 6.0)	2.9 (1.2, 5.5)	2.7 (1.7, 4.1)	2.8 (1.6, 4.6)	3.7 (1.8, 4.7)	2.8 (1.6, 4.7)
Monitoring strategy, N (%)^a^							
Clinical only	0 (0.0)	0 (0.0)	0 (0.0)	0 (0.0)	0 (0.0)	126 (10.5)	126 (3.5)
Routine CD4	0 (0.0)	55 (44.7)	0 (0.0)	0 (0.0)	0 (0.0)	520 (43.4)	575 (16.2)
Routine CD4 + targeted VL	0 (0.0)	0 (0.0)	3 (0.7)	0 (0.0)	0 (0.0)	399 (33.3)	402 (11.3)
Routine CD4 + routine VL	72 (100.0)	68 (55.3)	461 (99.4)	442 (100.0)	1255 (100.0)	154 (12.8)	2452 (69.0)
AIDS diagnosis, N (%)							
None	63 (87.5)	114 (92.7)	354 (76.3)	218 (49.3)	463 (36.9)	560 (46.7)	1772 (49.9)
Prior to start of first‐line	7 (9.7)	7 (5.7)	86 (18.5)	164 (37.1)	784 (62.5)	402 (33.5)	1450 (40.8)
Between first‐ and second‐line	2 (2.8)	2 (1.6)	24 (5.2)	60 (13.6)	8 (0.6)	237 (19.8)	333 (9.4)
WHO immune status, N (%)^b^							
None	25 (44.6)	31 (29.3)	183 (46.5)	96 (24.4)	496 (50.4)	160 (19.1)	991 (35.8)
Mild	18 (32.1)	9 (8.5)	55 (14.0)	42 (10.7)	137 (13.9)	72 (8.6)	333 (12.0)
Advanced	6 (10.7)	8 (7.6)	50 (12.7)	37 (9.4)	95 (9.7)	112 (13.4)	308 (11.1)
Severe	7 (12.5)	58 (54.7)	106 (26.9)	219 (55.6)	256 (26.0)	492 (58.9)	1138 (41.1)
CD4 count^c^							
N (%) with available measures	55 (76.4)	107 (87.0)	400 (86.2)	395 (89.4)	987 (78.7)	842 (70.2)	2786 (78.4)
Median (IQR)	828 (454, 1399)	239 (63, 661)	595 (330, 1046)	315 (127, 675)	577 (353, 884)	235 (81, 561)	445 (185, 818)
<200 cells/mm^3^, N (%)	2 (3.6)	51 (47.7)	46 (11.5)	141 (35.7)	117 (11.9)	374 (44.4)	731 (26.2)
>500 cells/mm^3^, N (%)	38 (69.1)	35 (32.7)	233 (58.3)	144 (36.5)	581 (58.9)	230 (27.3)	1261 (45.3)
CD4%^c^							
N (%) with available measures	56 (77.8)	72 (58.5)	382 (82.3)	370 (83.7)	936 (74.6)	615 (51.3)	2431 (68.4)
Median (IQR)	30 (21, 35)	20 (9, 30)	23 (15, 33)	14 (7, 22)	22 (15, 29)	13 (6, 21)	19 (11, 28)
HIV viral load^c^							
N (%) with available measures	53 (73.6)	56 (45.5)	414 (89.2)	323 (73.1)	1034 (82.4)	305 (25.4)	2185 (61.5)
≥1000 copies/mL, N (%)	44 (83.0)	47 (83.9)	290 (70.1)	298 (92.3)	808 (78.1)	296 (97.1)	1783 (81.6)
<1000 copies/mL, N (%)	9 (17.0)	9 (16.1)	124 (30.0)	25 (7.7)	226 (21.9)	9 (3.0)	402 (18.4)
WAZ^c^							
N (%)	51 (70.8)	119 (96.8)	342 (73.7)	366 (82.8)	934 (74.4)	1141 (95.2)	2953 (83.1)
Median (IQR)	−0.4 (−1.2, 0.5)	−1.5 (−2.7, −0.6)	−0.1 (−0.9, 0.7)	−1.9 (−3.0, −0.9)	−1.2 (−2.1, −0.4)	−2.0 (−3.0, −1.1)	−1.5 (−2.5, −0.5)

ART, antiretroviral treatment; IQR, interquartile range; SSA, sub‐Saharan Africa; VL, HIV viral load; WAZ, weight‐for‐age z‐score; WHO, World Health Organization.

^a^ This is a cohort‐level variable, derived from the frequency and availability of CD4 and VL measures across all ART‐treated children as previously described. [12]

^b^ none: CD4%> 35% for those <12 months of age,> 30% for those 12 to 35 months of age, >25% for those 36 to 59 months of age, and a CD4 count of >500 cells/mm^3^ for those five years or older; Mild: CD4% of 30–35% for those < 12 months of age, 25% to 30% for those 12 to 35 months of age, 20–25% for those 36 to 59 months of age, and a CD4 count of 350 to 499 cells/mm^3^ for those five years or older; Advanced: CD4% of 25–29% for those < 12 months of age, 20–24% for those 12 to 35 months of age, 15% to 19% for those aged 36 to 59 months, and a CD4 count of 200 to 349 cells/mm^3^ for those five years or older; Severe: CD4% < 25% for those <12 months of age, <20% for those 12 to 35 months of age, <15% for those 35 to 59 months of age, and a CD4 count <200 cells/mm^3^ for those five years or older [21]

^c^ summary statistics calculated among those with available data.

While 69% of clinics met our definition for having routine CD4 and VL testing, this varied greatly across and within regions, with 13% of sites in SSA defined as having routine VL testing and 33% having targeted VL testing. At start of second‐line ART, 41% of children had severe immune deficiency and 50% ever had an AIDS‐defining diagnosis. Of note, severe immune deficiency at switch to second‐line ART was close to 60% in the rest of SSA as compared to 26% in Southern Africa, where 100% of clinic sites had routine CD4 and VL monitoring. This disparity in HIV progression on ART was also evidenced by the proportion of children who progressed to an AIDS diagnosis after start of first‐line ART and prior to switch to second‐line ART in SSA compared to Southern Africa (20% vs. 0.6% respectively). Of the children with available CD4 counts at switch to second‐line ART (78%), over a quarter had CD4 counts <200 cells/mm^3^, though this varied greatly across regions, from 3.6% of children in North America to 36% in Asia, 44% in SSA and 48% in Latin America. Of the 62% of children with VL measurements at start of second‐line, 82% had VLs > 1000 copies/mL. Median WAZs were below reference norms in all regions, with children in Asia and SSA frequently severely underweight at the start of second‐line ART (median (IQR): Asia, −1.9 (−3.0, −0.9); SSA, −2.0 (−3.0, −1.1)).

### 3.2 Second‐line ART regimens

**Figure 2:**
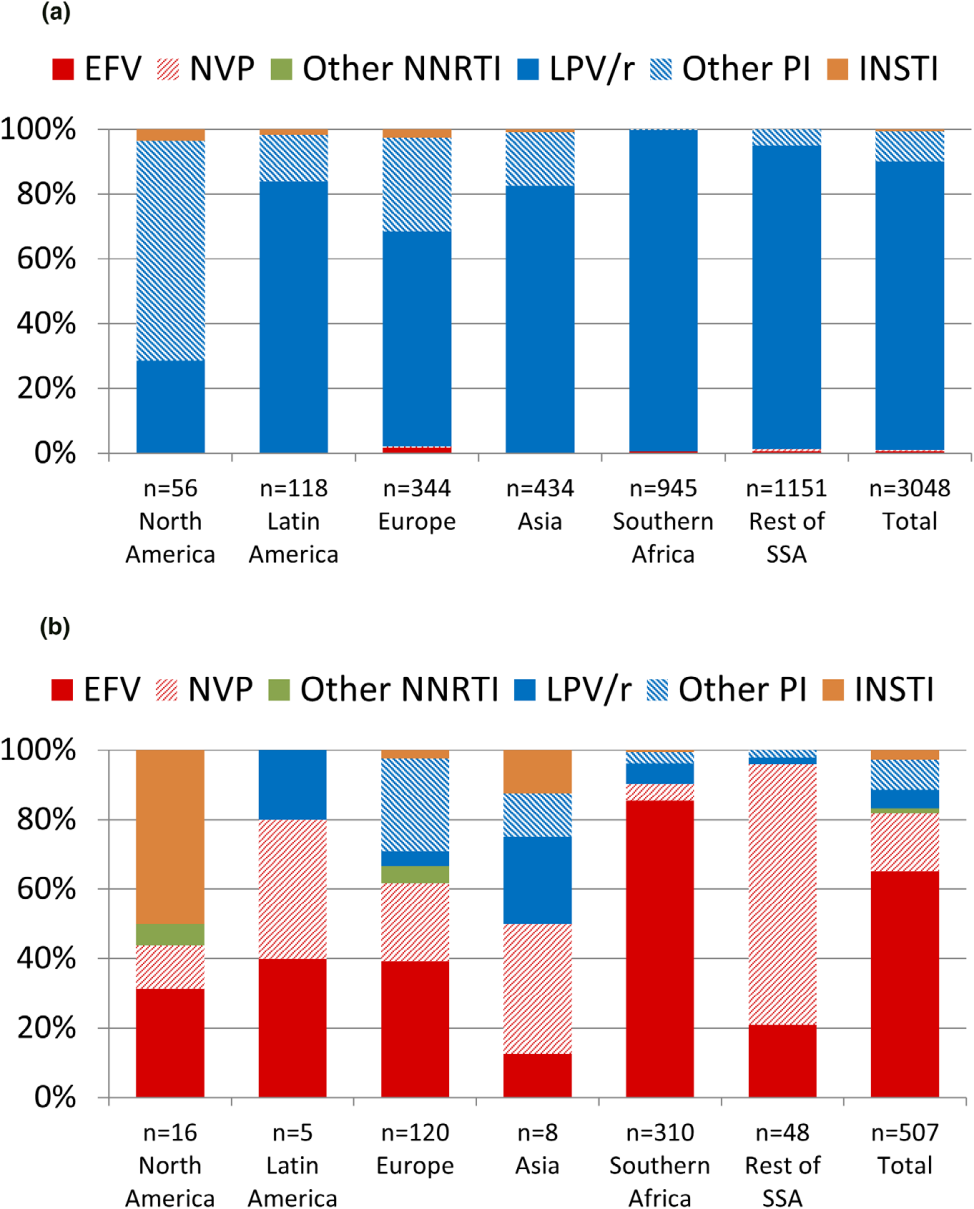
Second‐line ART regimens by region and first‐line ART regimen. **(a)** After an NNRTI‐based first‐line ART regimen. **(b)** After a PI‐based first‐line ART regimen. ART, antiretroviral therapy; EFV, efavirenz; INSTI, integrase strand transfer inhibitor; LPV/r, lopinavir/ritonavir; NNRTI, non‐nucleoside reverse transcriptase inhibitor; NVP, nevirapine; PI, protease inhibitor; SSA, sub‐Saharan Africa.

Among the 86% of participants who were on an NNRTI‐based first‐line regimen, the majority (89%) initiated an LPV/r‐based second‐line regimen, particularly in Southern Africa (99%) and SSA (94%) (Figure 2a). In the other regions, second‐line regimens with alternate PIs (e.g. atazanavir, darunavir) or INSTIs (e.g. raltegravir) were more prevalent, particularly in North America where 67% and 4% of children started a second‐line regimen with an alternate PI or INSTI respectively. Among the 14% of children who were on a PI‐based first‐line regimen, the majority (65%) initiated a second‐line ART with EFV, though second‐line regimens with NVP were more prevalent in SSA (75%) (Figure 2b).

### 3.3 Follow‐up and outcomes after switch to second‐line ART

**Figure 3:**
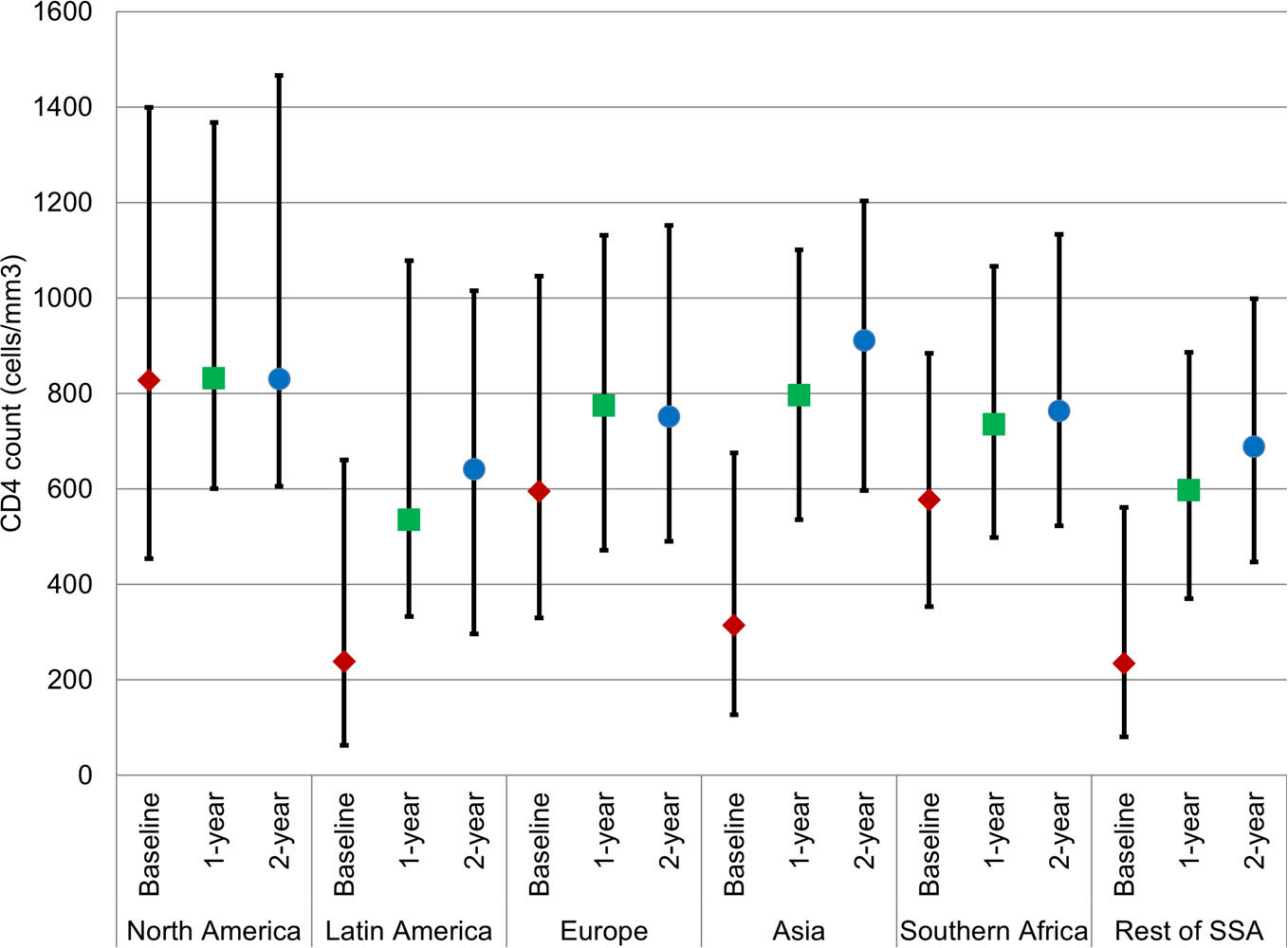
Median CD4 counts with interquartile ranges over two years of follow‐up by region.

Observed years of follow‐up after switch to second‐line ART ranged by region, from a median (IQR) of 1.8 (0.7, 3.3) in SSA, 2.2 (1.0, 4.0) in Southern Africa, 3.4 (1.9, 5.3) in Asia, 3.7 (2.1, 5.9) in Latin America, 4.0 (2.1, 6.8) in Europe, to 5.3 (2.7, 8.4) in North America. Children in almost all regions experienced improvements in median CD4 counts from switch to second‐line ART at one and two years of follow‐up, with more dramatic improvements in the regions with lower CD4 counts at start of second‐line (e.g. Latin America, Asia, SSA) (Figure 3). In North America, median CD4 count at the start of second‐line ART was in the upper range of normal and stayed stable during two years of follow‐up after switch to second‐line. Overall improvements in median CD4 counts from switch to second‐line ART were 180 cells/mm^3^ (IQR: −11, 422) and 199 cells/mm^3^ (IQR: −41, 496) at one and two years of follow‐up respectively. Median CD4 counts were >500 cells/mm^3^ across all regions at one and two years after switch to second‐line ART (Tables S1 and S2). Of note, CD4 measurements were available for over 75% of children in all regions across all time points including at switch to second‐line ART, except for SSA where CD4 count availability ranged from 69% to 70% across all time points. In additional subgroup analyses, there were increases in median CD4 counts over two years after switch across CD4 count categories at switch, age categories at switch, WAZ categories at switch, and second‐line regimen type, with more substantial increases among subgroups with lower CD4 counts at switch to second‐line ART (Figure S2).

In contrast to CD4 counts, little change in WAZ was observed in any region after switch to second‐line ART (Figure S3). There were also no substantial improvements in WAZ over two years after switch by CD4 count at switch, age at switch and second‐line regimen type, though there were some small improvements over time among children with CD4 counts <500 cells/mm^3^, children who were <10 years of age, and children who initiated PI‐ or NNRTI‐based second‐line ART. More substantial improvements in WAZ were observed over time among children who were severely wasted at switch (i.e. WAZ <−3), though median WAZ was still less than three standard deviations below the mean two years after switch (Figure S4).

**Figure 4:**
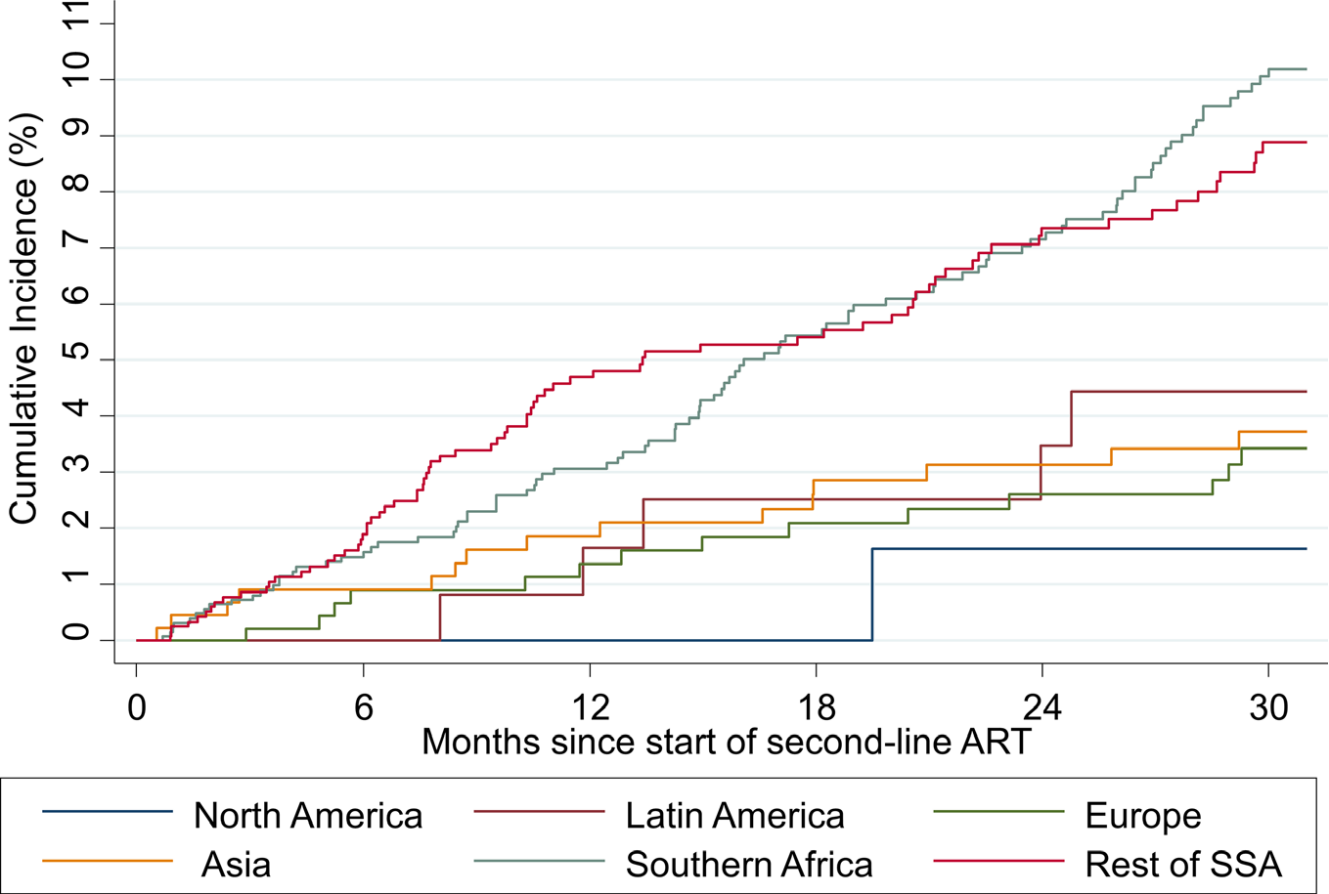
Cumulative incidence of loss to follow‐up by region.

**Figure 5:**
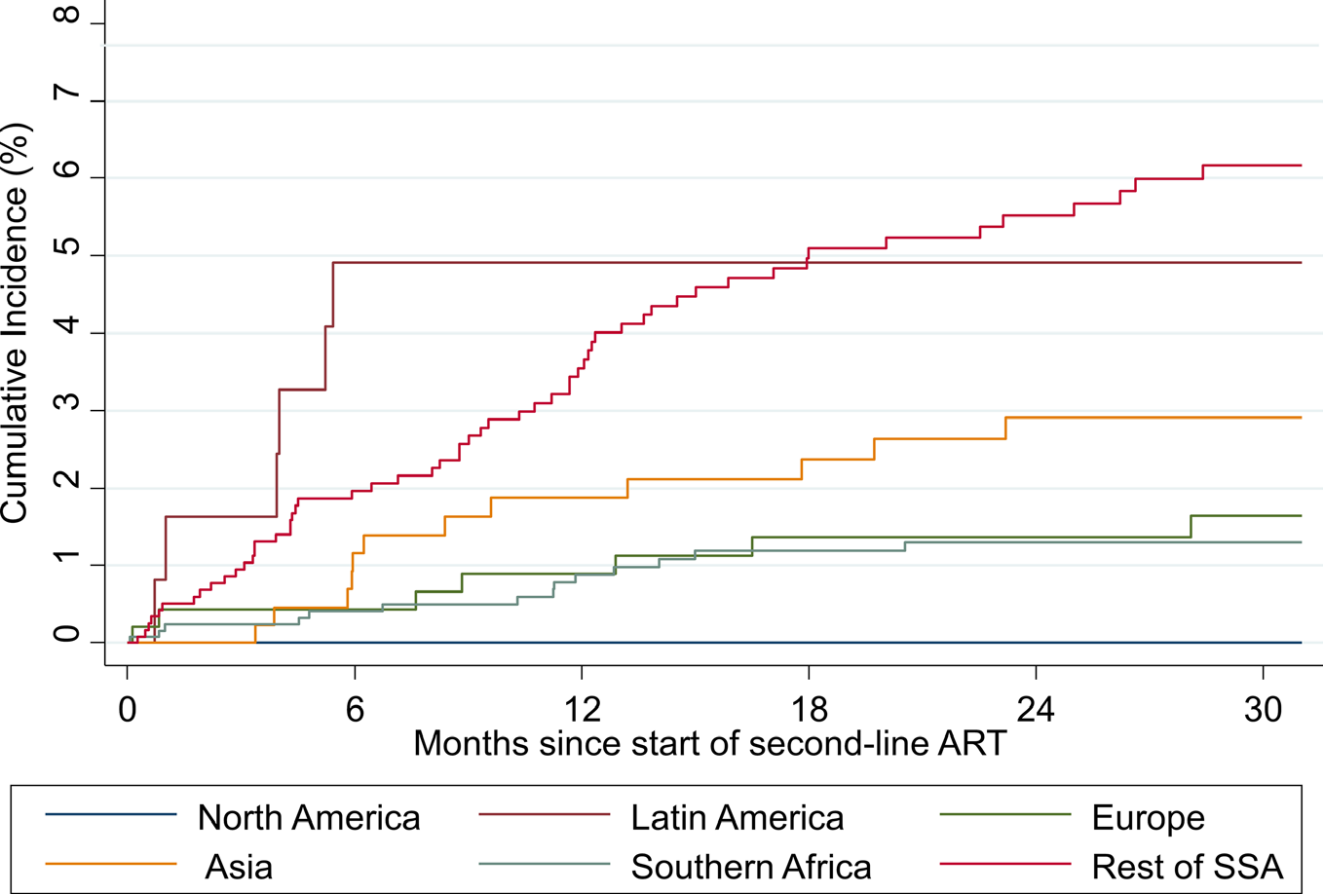
Cumulative incidence of mortality by region.

The cumulative incidence of LTFU at two years was <5% in all regions except Southern Africa and SSA, where it was 7.1% (95% confidence interval (CI): 5.7%, 8.8%) and 7.4% (95% CI: 5.8%, 9.2%) respectively (Figure 4). The cumulative risk of mortality was less than 3% at two years in North America, Europe, Asia and Southern Africa (Figure 5). In the first six months after second‐line ART, the risk of mortality was 4.9% (95% CI: 2.0, 9.8) in Latin America, with no additional deaths over follow‐up, and 5.5% (95% CI: 4.2, 7.1) in SSA at two years. Among the 92 children who were reported to have died within two years of switch to second‐line ART, their median age at switch was 8.2 years (IQR: 5.2, 12.8). 85/92 (92.4%) had initiated a PI‐based second‐line regimen, 5/92 (5.4%) an NNRTI‐based regimen and 2/92 (2.2%) an INSTI‐based regimen. The median CD4 count, CD4% and WAZ at switch to second‐line among the subgroup who died within two years after switch and with available measures were 90 cells/mm^3^ (IQR: 23, 377; n:74), 5% (IQR: 2, 16; n: 59) and −3.8 (IQR: −5, −1.9; n:85) respectively.

## 4 Discussion

We describe the largest cohort of children living with HIV on second‐line ART to date, and specifically include the largest population of children on second‐line ART from Africa, where HIV disease burden is highest. Almost 70% of children in the analysis were from Southern Africa and the rest of SSA.

Our study reports on the cumulative incidences of LTFU and mortality among children on second‐line ART. We observed low to moderate risks of LTFU and mortality across all regions, though higher risks were observed among children in Southern Africa and SSA, which is consistent with prior studies evaluating ART outcomes in these regions [27]. It is important to note that this cohort reflects children living with HIV who have survived for long enough to access both first‐ and second‐line ART. Moreover in many resource‐limited settings, switching to second‐line is less likely to occur if providers are unable to identify children as failing treatment or are uncomfortable switching them to second‐line regimens which are often more difficult to adhere to. Thus, this cohort reflects a group of paediatric survivors who have had consistent and reliable engagement with clinical care over the course of their treatment and who have thus had better potential for medication adherence or access to clinical management supports such as adherence counselling before the time of ART switch.

At two years of follow‐up, we observed improvements in CD4 counts similar to what have been reported in previous studies conducted primarily in Asia [14, 16, 18, 19, 20, 21]. While it is reassuring to observe immune recovery after switch to second‐line ART, we noted a large proportion of children with severe immune deficiency and AIDS progression at the time of switch to second‐line ART in some settings. This suggests that recognition of treatment failure and transition to second‐line regimens is often very late. While it is true that second‐line ART became available in later calendar years in settings such as SSA, our findings suggest a need for improved recognition and management of treatment failure in children.

In contrast to CD4 count, we did not observe improvements in WAZ over two years of follow‐up after switch to second‐line ART. This suggests that second‐line ART is not necessarily associated with shorter‐term improvements in WAZ among children with perinatal HIV infection. One other study also reported no improvements in WAZ after 96 weeks on second‐line ART [19]. Many children therefore remained severely underweight in some settings, which has implications for future cognitive development and mortality [28, 29]. More data are needed to understand why we did not observe gains in weight after switch to second‐line ART.

While the incidence of switch to second‐line among children globally is overall very low after initiation of ART [12], the current generation of children who switched to second‐line ART did so after a median of less than three years on first‐line ART and at a median of 8.4 years of age. Previous studies of children on PI‐based second‐line ART have reported a low prevalence of major resistance mutations to second‐line agents [15, 21], suggesting that these regimens should be efficacious in the long‐term with appropriate adherence support. However, third‐line and fourth‐line options will become a necessity with lifelong therapy. Ensuring access to new, better tolerated antiretroviral drugs and supporting medication adherence will be continuing and important needs.

While our study represents a large global analysis, we could not effectively elucidate switches to second‐line ART due to treatment failure because we could not evaluate risks of virologic failure across regions due to varying VL monitoring strategies and testing access. Particularly in regions where targeted VL monitoring is prevalent, it would be difficult to interpret risks of virologic failure as children with clinical signs of disease progression would be more likely to receive a VL test. For our primary outcomes of interest, CD4 count and WAZ, the proportion of missing data ranged from 6% to 31% and 3% to 29% respectively, across regions and time points. Our data, however, is reflective of routine clinical care and the contexts in which treatment decisions are being made for children living with HIV globally. Given the heterogeneity in available data, we also did not report on height measures or calculate body mass index, limiting our ability to fully understand growth responses after switch to second‐line. The expansive calendar time span of the study, while reflective of the regional disparity in when children began enrolling in HIV care services, also added to the heterogeneity of our data with respect to available and recommended second‐line ART options and disease monitoring strategies across calendar time and region. We were also unable to evaluate longer‐term outcomes after switch to second‐line ART across all regions as second‐line ART became available in later calendar years in SSA, thus shortening the available follow‐up time. Lack of access to new ART drugs and limited follow‐up in some regions also restricted our ability to define switch to third‐line ART and thus durability of second‐line. Our observations on the outcomes after switch to second‐line ART, however, can inform need for third‐line ART in specific regions.

## 5 Conclusions

Children living with perinatal HIV who switched to second‐line ART in our global cohorts have responded well, with increases in CD4 counts and low to moderate rates of LTFU and mortality within two years after switch. The large proportion of children with severe immune deficiency at time of switch in some settings emphasizes the need for improved recognition and management of treatment failure in children. The early age at switch among children and adolescents on second‐line ART, also emphasizes the importance of providing access to more durable regimens to preserve their long‐term treatment options and health through adulthood.

## Competing Interest

The authors declare no competing interests.

## Authors’ contributions

All authors listed below as belonging to the Project Team, Data Coordinating Team and the Writing Team have contributed sufficiently to the conception, design, data collection, analysis, writing and/or review of the manuscript to take public responsibility for it. All authors have also read and approved the final manuscript.

## The cipher global cohort collaboration

**Project Team:** Kunjal Patel* (Harvard T. H. Chan School of Public Health, Boston, Massachusetts, United States of America), Colette Smith^†^ (MRC Clinical Trials Unit at University College London, London, United Kingdom), Intira Jeannie Collins^†^ (MRC Clinical Trials Unit at University College London, London, United Kingdom), Ruth Goodall^†^ (MRC Clinical Trials Unit at University College London, London, United Kingdom), Elaine J Abrams (ICAP at Columbia University, Mailman School of Public Health, and Vagelos College of Physicians & Surgeons, Columbia University, New York, New York, United States of America), Annette H Sohn (TREAT Asia/amfAR, The Foundation for AIDS Research, Bangkok, Thailand), Thahira J Mohamed (Pediatric Institute, Hospital Kuala Lumpur, Kuala Lumpur, Malaysia), Russell B Van Dyke (Tulane University School of Medicine, New Orleans, Louisiana, United States of America), Pablo Rojo (Hospital Universitario 12 de Octubre, Madrid, Spain), Kara Wools‐Kaloustian (Indiana University School of Medicine, Indianapolis, Indiana, United States of America), Jorge Pinto (Federal University of Minas Gerais, Belo Horizonte, Brazil), Andrew Edmonds (Gillings School of Global Public Health, The University of North Carolina at Chapel Hill, Chapel Hill, North Carolina, United States of America), Irene Marete (Moi University, Nairobi, Kenya), Mary Paul (Baylor International Pediatric AIDS Initiative, Texas Children’s Hospital, Houston, Texas, United States of America), Harriet Nuwaqaba‐Biribonwoha (ICAP at Columbia University, and Mailman School of Public Health, New York, New York, United States of America), Valériane Leroy (Inserm (French Institute of Health and Medical Research), UMR 1027 Université Toulouse 3, Toulouse, France), Mary‐Ann Davies (Center for Infectious Diseases Epidemiology and Research, University of Cape Town, Cape Town, South Africa), and Rachel Vreeman* (Icahn School of Medicine at Mount Sinai, New York, New York, United States of America). *Project co‐chairs; ^†^Project statisticians/epidemiologists.

**Data Coordinating Team:** Nicky Maxwell (University of Cape Town, Cape Town, South Africa),

Venessa Timmerman (University of Cape Town, Cape Town, South Africa), Charlotte Duff (MRC Clinical Trials Unit at University College London, London, UK).

**Writing Team:** *CIPHER Executive Committee:* Lynne Mofenson, Linda‐Gail Bekker, Marissa Vicari, Shaffiq Essajee, Martina Penazzato; *CIPHER Project Oversight Group:* Intira Jeannie Collins, Kara Wools‐Kaloustian, Mary‐Ann Davies, Valériane Leroy, Ruth Goodall, Kunjal Patel, Colette Smith, Rachel Vreeman, Amy Slogrove, Paige Williams, Siobhan Crichton, George Seage III; *BIPAI:* Lineo Thahane (Baylor College of Medicine Children's Foundation Lesotho), Peter N Kazembe (Baylor College of Medicine Children's Foundation Malawi), Bhekumusa Lukhele (Baylor College of Medicine Children’s Foundation‐eSwatini), Lumumba Mwita (Baylor College of Medicine Children's Foundation‐Tanzania), Adeodata Kekitiinwa‐Rukyalekere (Baylor College of Medicine Children's Foundation – Uganda), Sebastian Wanless (Baylor International Pediatric AIDS Initiative at Texas Children’s Hospital, Data Manager), Mogomotsi S Matshaba (Botswana‐Baylor Children's Clinical Centre of Excellence); *EPPICC:* Intira Jeannie Collins (MRC Clinical Trials Unit at University College London, London, UK), Ruth Goodall (MRC Clinical Trials Unit at University College London, London, UK), Colette Smith (Faculty of Population Health Sciences, University College London, London, UK), Tessa Goetghebuer (Hospital St Pierre, Brussels, Belgium), Claire Thorne (UCL Great Ormond Street Institute of Child Health, University College London, UK), Josiane Warszawski (INSERM, France), Luisa Galli (Università degli Studi di Firenze, Italy), Sybil Geelen (Wilhelmina Children’s Hospital, University Medical Centre Utrecht, University of Utrecht, Utrecht, The Netherlands), Diana M Gibb (MRC Clinical Trials Unit at University College London, London, UK), Carlo Giaquinto (Padova University/ PENTA Foundation, Italy), Magdalena Marczynska (Medical University of Warsaw, Hospital of Infectious Diseases in Warsaw, Poland), Laura Marques (Centro Hospitalar do Porto, Portugal), Filipa Prata (Hospital de Santa Maria, Lisbon, Portugal), Luminita Ene (Victor Babes Hospital, Bucharest, Romania), Liubov Okhonskaia (Republican Hospital of Infectious Diseases, St Petersburg, Russian Federation), Pablo Rojo (Hospital Doce de Octubre, Madrid), Antoni Noguera‐Julian (Hospital Sant Joan de Déu, Universitat de Barcelona, Barcelona), Lars Naver (Karolinska University Hospital), Christoph Rudin (University Children’s Hospital, Basel), Gonzague Jourdain (Faculty of Associated Medical Sciences, Chiang Mai University and the Institut de recherche pour le développement, France), Ali Judd (MRC Clinical Trials Unit at University College London, London, UK), Alla Volokha (Shupyk National Medical Academy of Postgraduate Education, Kiev); *IeDEA‐CCASAnet:* Jorge Pinto (Department of Pediatrics, School of Medicine, Federal University of Minas Gerais, Brazil), Vanessa Rouzier (GHESKIO Center, Port‐au‐Prince, Haiti), Regina Succi (Universidade Federal de São Paulo, Brazil); *IeDEA Asia‐Pacific:* Kulkanya Chokephaibulkit (Siriraj Hospital, Mahidol University, Bangkok, Thailand), Annette H Sohn (TREAT Asia/amfAR, Bangkok, Thailand), Azar Kariminia (Kirby Institute, University of New South Wales, Sydney, Australia), Thahira J Mohamed (Pediatric Institute, Hospital Kuala Lumpur, Kuala Lumpur, Malaysia); *IeDEA Central Africa:* Marcel Yotebieng (College of Public Health, Ohio State University, Columbus, USA), Andrew Edmonds (Gillings School of Global Public Health, University of North Carolina at Chapel Hill, USA), Patricia Lelo (Pediatric Hospital Kalembe Lembe, Lingwala, Kinshasa, Demogratic Republic of Congo); *IeDEA East Africa:* Rita Lyamuya (Morogoro Regional Hospital, Morogoro, Tanzania), Irene Marete (Academic Model Providing Access to Healthcare (AMPATH), Eldoret, Kenya), Patrick Oyaro (Family AIDS Care and Education Services (FACES), Kenya Medical Research Institute (KEMRI), Kisumu, Kenya), Kara Wools‐Kaloustian (Indiana University School of Medicine, Department of Medicine, Division of Infectious Diseases, Indianapolis, Indiana); IeDEA Southern Africa: Andrew Boulle (Center for Infectious Disease Epidemiology and Research, University of Cape Town, South Africa, Khayelitsha ART Programme, Cape Town, South Africa; Westen Cape Department of Health, Cape Town, South Africa), Kennedy Malisita (Queen Elisabeth Central Hospital, Blantyre, Malawi), Geoffrey Fatti (Kheth'Impilo, Cape Town, South Africa, Division of Epidemiology and Biostatistics, Department of Global Health, Faculty of Medicine and Health Sciences, Stellenbosch University, South Africa), Andreas D Haas (Institute of Social and Preventive Medicine, University of Bern, Switzerland), Mary‐Ann Davies (Center for Infectious Diseases Epidemiology and Research, University of Cape Town, Cape Town, South Africa); IeDEA West Africa: Sophie Desmonde (Inserm 1027, Université de Toulouse 3, Toulouse, France), Fatoumata Dicko (CHU Gabriel Touré, Bamako, Mali), Valériane Leroy (Inserm (French Institute of Health and Medical Research), UMR 1027 Université Toulouse 3, Toulouse, France); *IMPAACT/PHACS:* Mark J Abzug (University of Colorado School of Medicine and Children’s Hospital Colorado, USA), Paige Williams (Harvard T. H. Chan School of Public Health, USA), Murli Purswani (Bronx‐Lebanon Hospital Center, USA), George Seage III (Harvard T. H. Chan School of Public Health, USA), Russell Van Dyke (Tulane University, USA), Ellen Chadwick (Feinberg School of Medicine, Northwestern University, USA); *ICAP‐Optimal Models:* Elaine Abrams (ICAP‐Columbia University, Mailman School of Public Health, USA), Chloe Teasdale (ICAP‐Columbia University, Mailman School of Public Health, USA), Harriet Nuwagaba (ICAP‐Columbia University, Mailman School of Public Health, USA).

## Supporting information


**Table S1.** Clinical and laboratory outcomes at one year of follow‐up after start of second‐line ART
**Table S2.** Clinical and laboratory outcomes at two years of follow‐up after start of second‐line ART
**Figure S1.** Study population derivation.
**Figure S2.** Median CD4 counts (cells/mm^3^) with interquartile ranges over two years of follow‐up by characteristics at switch to second‐line.
**Figure S3.** Median weight‐for‐age z‐scores with interquartile ranges over two years of follow‐up by region.
**Figure S4.** Median weight‐for‐age z‐scores with interquartile ranges over two years of follow‐up by characteristics at switch to second‐line.
